# Overview of techniques to manage shoulder dystocia during vaginal birth

**DOI:** 10.18332/ejm/142097

**Published:** 2021-10-20

**Authors:** Anastasia Bothou, Dimitra-Maria Apostolidi, Panagiotis Tsikouras, Georgios Iatrakis, Aggeliki Sarella, Dimitrios Iatrakis, Panagiotis Peitsidis, Aggeliki Gerente, Xanthoula Anthoulaki, Nikolaos Nikolettos, Stefanos Zervoudis

**Affiliations:** 1 Department of Midwifery, School of Health and Care Sciences, University of West Attica, Athens, Greece; 2 Department of Obstetrics and Gynecology, Health Sciences School, Democritus University of Thrace, Alexandroupolis, Greece; 3 REA Maternity Hospital, Athens, Greece; 4 National Technical University of Athens, Athens, Greece

**Keywords:** shoulder dystocia, interventional delivery, McRoberts’ maneuver, Wood’s maneuver, delivery of the posterior arm, axillary traction

## Abstract

Shoulder dystocia is an obstetric emergency which is unpredictable and complicates approximately 0.5–1% of vaginal births. This article discusses the risk factors and the associated fetal and maternal complications, while it is also an overview of techniques and algorithms to handle shoulder dystocia.

## INTRODUCTION

### Definition and etiology

Shoulder dystocia is an unpredictable obstetric emergency^1-12^ and is related with neonatal morbidity and mortality and in some severe cases with maternal complications^1,4,13-18^. It is defined as a vaginal birth that requires special maneuvers after the neonate’s head has been delivered and the birth of the fetal shoulders has failed^2,19^. Shoulder dystocia complicates approximately 0.5– 1% of vaginal births^1-3,13,19,20^. Nowadays, this percentage is increasing because there are bigger neonates being born than in the past and possibly due to the increased use of epidural anesthesia. Furthermore, the percentages may be different depending on the size of the fetus. Specifically, the likelihood for shoulder dystocia is about 1% for fetuses weighing <4 kg, 5% for fetuses 4–4.5 kg, and about 10% for fetuses weighing >4.5 kg^18^.

### Risk factors

Macrosomia of the fetus is the most significant risk factor for shoulder dystocia^2,4,6,13,15,19,21-23^, however other risk factors are also responsible, for example: maternal diabetes^2,4,6,19,21,24^, previous history of shoulder dystocia^2,6,19,20,25^, pelvic stenosis, large maternal weight gain^20,26^ or maternal obesity^2,6,19,20^, male gender, multiparity, assisted vaginal delivery with vacuum or forceps, prolonged second stage, and >42 weeks of pregnancy etc. Only 50–70% of the risk factors can predict shoulder dystocia, except of gestational diabetes^2,19^. Moreover, despite fetal macrosomia being the most significant factor for shoulder dystocia, >50% of the cases occur in pregnancies with a normal birth weight fetus.

Shoulder dystocia should be differentiated from breech delivery and umbilical cord prolapse and ‘delay of shoulder delivery with normal head rotation’^27^.

### Complications

Shoulder dystocia is associated with a variety of complications for the fetus such as: paresis of brachial plexus^2,4,11,15,16,19,20,28-31^, perinatal asphyxia^2,11,16,19,30,31^, hypoxic-ischemic encephalopathy^2,19^, humerus fracture^27^, clavicular fracture^2,11,15,19,31^, and perinatal mortality^2,19,30^. Furthermore, soft tissue injuries are the most common maternal complication, with an increased rate of third- and fourth-degree lacerations or tears in the vagina and vulva.

## CLINICAL MANAGEMENT

**Figure 1:**
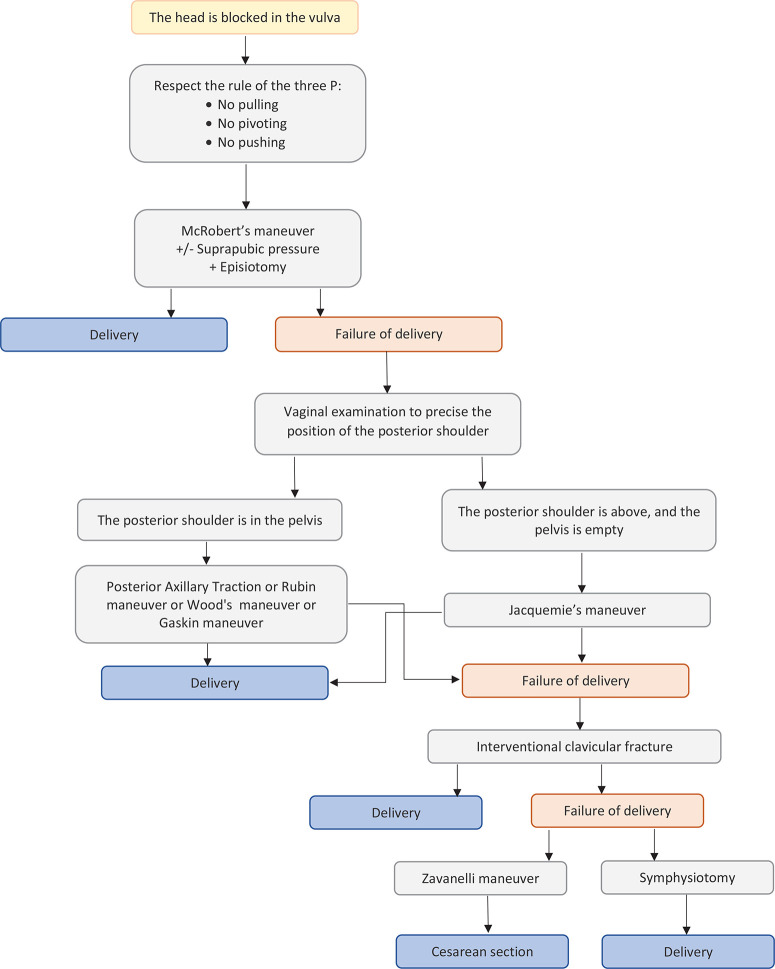
Algorithms for the management of shoulder dystocia

Midwives and obstetricians should be able to deal with shoulder dystocia at any time^6,10,32^. This means that they are adequately and distinctly trained^1^ through simulation^3,4,33^ so that they know how to perform obstetric maneuvers^2,5,10,19^ and apply the protocols^1,3,5^. The main aim is to reduce the birth interval between the head and body in order to reduce the risk of birth asphyxia and to deliver the neonate without damaging the brachial plexus by pulling the neck^7^. When shoulder dystocia has appeared, it is of great importance that the physician recognizes the situation and immediately asks for help from other team members. All the known maneuvers are typically divided into simple and complicate maneuvers that are described below. It is noteworthy that the recommendation about the appropriate amount of time to spend on each maneuver is up to 30 seconds. Figure 1 describes the algorithms for the management of shoulder dystocia.

### First-line maneuvers

*McRoberts’ maneuver*

This maneuver was introduced in 1983^34^. It is a simple but effective maneuver^35,36^ of vaginal delivery where the patient is in a lithotomy position with abducted legs bending the knees at the height of the abdomen. Through this, the pelvic diameters are increased to the maximum, the anterior shoulder due to the straightening of the sacrum and the movement of the posterior shoulder over the sacrum descends below the pubic symphysis and the posterior shoulder descends lower into the pelvis^36-38^. However, McRoberts’ is not always successful. In case of traction of the head there is high risk of complications such as brachial plexus injury of the newborn (BPI)^37,39^. Moreover, lower extremity neuropathy due to prolonged compression of the femoral nerve of the mother and complications such as hip stiffness and extension of the knee to the site of injury could occur^36^.

*Suprapubic pressur*

McRoberts’ is often combined with suprapubic pressure^18,19,35,38,39^ where a second health-worker applies pressure with a palm or fist down and sideways to move the anterior shoulder below the pubic symphysis and towards the oblique diameter of the pelvis, which is the largest diameter. It is recommended in cases of mild shoulder dystocia^18,35^. Interestingly, the American College of Obstetricians and Gynecologists’ current recommendation is to begin with this maneuver combined with suprapubic pressure. In fact, the success of the aforementioned combination ranges 24–62%^34^.

*Jacquemier’s maneuver*

This maneuver consists of delivery of the whole posterior arm followed by posterior shoulder delivery^40^; it is recommended when the McRoberts maneuver combined with suprapubic pressure fails and it is not a primary maneuver^18,35^. In general, because of the difficulty of the maneuvers and the pain to the woman, an epidural anesthesia is mandatory. The obstetrician inserts a hand into the vagina behind the posterior fetal shoulder to grasp the fetal elbow and bend it to the fetal chest. Then, with gentle traction, the fetal elbow is delivered followed by the delivery of the posterior shoulder. If this fails, it is recommended to rotate the fetus internally so that the anterior shoulder is now posterior and then repeated. However, this has been related to complications such as humerus fracture, especially when flexion of the elbow is impossible or difficult^17,18,35^.

*Wide episiotomy*

Wide episiotomy is necessary to be performed to facilitate the procedures^7,19,41-43^. More specifically, the maneuver of Rubin–Wood needs more space in order to be efficient^7,12,19,35,37,39,42^. This procedure is included in most algorithms such as the Hernandez & Wendel and the ALARMER and, placed as an obligatory procedure before the internal maneuvers, except in the HELPERR algorithm^7,9^. In summary, the majority of authors recommend to perform a wide episiotomy.

*Gaskin maneuver (‘all-fours’ position)*

This maneuver is inspired from the traditional procedures of dystocic deliveries in Guatemala. The mother is placed in a knee-hands position^7,27,35,37^ or in a sprinter position^35,41^ but never in a knee-chest position^7,35^. In this way the cavity of the sacrum increases and the gravity in combination with the traction of the posterior or anterior shoulder facilitates the release of the shoulder^27,35^. However, this treatment is not indicated in cases of epidural or dorsal anesthesia^7,35^.

### Second-line maneuvers

*Posterior axillary traction*

Posterior axillary traction is recommended as a second internal maneuver if the delivery of the posterior shoulder fails^18,35,41^ which, according to Ansell et al.^17^, appears to have better results and is thus recommended to be used as the first internal maneuver if McRoberts’ with suprapubic pressure fails^37^. The health worker places a hand into the posterior aspect of the pelvis and with the thumb and first finger grasps the posterior shoulder around the axilla, and with the middle finger applies traction only through the axilla. As a result, the posterior shoulder is delivered followed by the delivery of the anterior arm^18,35,37^.

*Rubin maneuver*

The Rubin maneuver (rotation of the shoulder) was first described in 1964^34^. It involves rotating the shoulders to the oblique diameter through suprapubic pressure^7,35,37^. If this fails, Rubin II is recommended. The physician inserts a hand into the vagina after anesthesia and applies pressure to the anterior aspect of the most accessible shoulder in order to reduce the bisacromial diameter^7,27^ then rotates the shoulder^27,35,37^. Clinical studies have shown that the Rubin maneuver is associated with fewer complications and requires less traction compared to the McRoberts maneuver^7,35^. However, it is more invasive than the McRoberts maneuver and less easily performed in patients without anesthesia^35^.

*Wood’s maneuver*

Wood’s maneuver (rotation of the fetus) is the second internal maneuver after Rubin’s and these two are quite often combined^7,35,37^. It was first described in 1942^34^. This maneuver puts pressure on the front clavicle surface of the posterior shoulder so that the fetus rotates 180 degrees and the front shoulder is released^35,37^. If combined with the Rubin maneuver then pressure is applied to both shoulders clockwise or counter clockwise to rotate the baby^7,35^.

### Third-line maneuvers

*Intentional clavicular fracture*

This is achieved by applying pressure to the clavicle of the fetus, when more conservative approaches fail^7,27^. This technique reduces the bisacromial diameter, but the clinician must be very careful so not to injure the underlying vascular fracture or even the lung fracture^27,35^.

*Zavanelli maneuver*

When all techniques have failed, then the Zavanelli maneuver is suggested^2,19,37^. This maneuver came into popular use in the early 1980s. The mother receives terboutaline sc or some other uterine relaxant^7,35^. The fetal head should then be turned in the anterior occipital position, flexed from the extended position and then pushed back into the pelvis. A cesarean section is performed immediately^7,27,35,37^. During the procedure it is mandatory to monitor the fetal heart rate^35^.

*Symphysiotomy*

Symphysiotomy is only recommended when all other techniques have failed^7,27,35,37^. In fact, Menticoclou^18^ states that it should be applied only after 5 minutes if the dystocia has not been solved yet and the other maneuvers, even the Zavanelli, have failed. It has been used as a last resort. It involves the surgical division of the fibrous tissue and cartilage^7^ of the pubic symphysis^7,35,37^ in order to increase the pelvic diameters^7,35^. However, it should be avoided because the separation of the pubic symphysis is not restored and has been related to complications such as bladder, urethral and vaginal injury. These injuries could lead later to urinary incontinence, chronic pelvic pain, and unstable pelvis^7,35,37^.

### Algorithms

Although Gottlieb and Galan^6^ consider that there is no specific algorithm for the management of shoulder dystocia, most researchers suggest various algorithms.

*The HELPERR*

The HELPERR^7,25^ algorithm is as follows: Help, Evaluate (for episiotomy), Legs (McRoberts’ position), Pressure (suprapubic), Enter (rotational maneuvers), Remove (posterior arm), and Roll (hands and knees).

*The Hernandez & Wendel*

The Hernandez & Wendel algorithm involves: 1) Call for help, 2) Wide perineotomy, 3) Suprapubic pressure, 4) McRoberts’ maneuver, 5) Delivery of the posterior arm, and 6) Wood’s screw maneuver or Rubin maneuver.

*The ALARMER*

The ALARMER algorithm when the episiotomy in contrast to HELPERR is in 6th place^7^.

*The British and French College*

The British College of Obstetricians and Gynecologists and the Collège National des Gynécologues et Obstétriciens Français^2^ propose the following algorithm: 1) Call for help, 2) No pushing, 3) McRoberts’ maneuver, 4) Suprapubic pressure and gentle head traction, 5) Wide perineotomy, 6) Jacquemier’s maneuver and Rubin’s or Wood’s maneuver, and if there is a failure, 7) Knee-elbow position, and repeat algorithm. If this also fails then the third-line maneuvers: clavicle fracture, Zavanelli maneuver, and symphysiotomy, are recommended^7,37^.

### Prevention

It is notable that cesarean section should be recommended in order to prevent shoulder dystocia only in the following cases: 1) fetus with weight >4.5 kg, if associated with maternal diabetes; 2) fetus with weight >5 kg and with an absence of maternal diabetes; 3) previous history of shoulder dystocia with severe maternal and neonatal complications; and 4) fetal macrosomia with a failure in progress to the second stage of delivery^2^. The main point is knowledge of the weight of the fetus, to avoid a difficult delivery including shoulder dystocia. This could be assessed by ultrasound which can estimate approximatively the weight of the fetus, but in some cases it can be over- or under-estimated. In case of very probable macrosomia, a cesarean section should be performed in order to avoid the difficult delivery of shoulder dystocia.

## CONCLUSION

Shoulder dystocia is an unpredictable obstetrics emergency and many maneuvers have been proposed for its management. The key to successful management is anticipation and suitable preparation. For this reason, both obstetricians and midwives should be able to perform all the obstetrics maneuvers and as quickly as needed in order to prevent potentially serious consequences. There is a need for an experienced team in this situation, on the other hand the maneuvers should be performed without losing time because each minute is crucial to avoid severe complications or death of the fetus.

## Data Availability

Data sharing is not applicable to this article as no new data were created.
